# Immediate Postoperative Activation of Vagus Nerve Stimulation (VNS) for Super-refractory Status Epilepticus: A Case Report

**DOI:** 10.7759/cureus.76509

**Published:** 2024-12-28

**Authors:** Karen J Camarena-Rubio, Brandon Flores-Patiño, Jonathan U Macías López, Diego Pichardo-Rojas, Valeria I Bravo Osorno, Irene Gomez-Oropeza, María F Castelo-Pablos, Sonia Iliana Mejía-Pérez, Elma Paredes, Manuel A Del Río-Quiñones, Laura Hernandez Vanegas

**Affiliations:** 1 Epilepsy Surgery, National Institute of Neurology and Neurosurgery, Mexico City, MEX; 2 Neurology, National Institute of Neurology and Neurosurgery, Mexico City, MEX; 3 Neurosurgical Oncology, National Institute of Neurology and Neurosurgery, Mexico City, MEX; 4 Epilepsy Clinic, National Institute of Neurology and Neurosurgery, Mexico City, MEX; 5 Clinical Research, National Institute of Neurology and Neurosurgery, Mexico City, MEX

**Keywords:** anti-nmda, encephalitis, immediate postoperative activation vns, super-refractory status epilepticus, vns

## Abstract

Anti-NMDA (N-methyl-D-aspartate) receptor encephalitis (ANRE) is a rare autoimmune condition targeting brain receptors, often linked to ovarian tumors in young women. In severe cases, it can lead to status epilepticus, but in sporadic cases, it may progress to super-refractory status epilepticus (SRSE), a dangerous state of continuous or repetitive seizures demanding urgent medical attention that continues or recurs more than 24 hours after the initiation of anesthetic therapy. We present a case report of anti-NMDA receptor limbic encephalitis-triggered SRSE terminated with vagus nerve stimulation (VNS) and titrated to high stimulation parameters in the immediate postoperative period. Titrating VNS to high stimulation parameters immediately postoperatively under specialized neuroanesthesia is a safe and effective treatment for nonconvulsive SRSE in anti-NMDA receptor limbic encephalitis. However, further research is needed to solidify this approach as a standard treatment option in these circumstances since the SRSE is rare. Expanding the evidence base will help improve patient outcomes and reduce morbidity and mortality associated with this condition.

## Introduction

Super-refractory status epilepticus (SRSE) occurs when seizures persist or recur after more than 24 hours of anesthetic treatment, constituting a severe neurological emergency with significant risks of morbidity and mortality. Approximately 23%-48% of patients with status epilepticus (SE) progress to RSE, and 22% of those with RSE advance to SRSE [1].

The most common causes of SRSE are an inadequate treatment of RSE or underlying factors such as infection, inflammation, or structural abnormalities [2]. A small retrospective study found that 47% of children with SRSE had immune-mediated encephalitis. For NMDA (N-methyl-D-aspartate), first-line therapies typically include a combination of corticosteroids, intravenous immunoglobulin (IVIG), and plasma exchange (PLEX). When first-line therapies have failed, a second-line option such as rituximab or cyclophosphamide is considered. In refractory cases, agents such as bortezomib or tocilizumab may be employed to target long-lived plasma cells [3].

Common treatments for SRSE include intravenous anesthetics, antiepileptic drugs, and alternative therapies such as ketogenic diet or vagus nerve stimulation (VNS) [1]. If these approaches fail, options such as brain surgery, perampanel, or inhalational anesthetics may be considered, although evidence remains limited. Although VNS has demonstrated efficacy in treating refractory epilepsy, its use in SRSE remains novel and is primarily based on case reports and small series. Despite the methodological limitations of these studies, the results suggest that acute VNS implantation could potentially interrupt SRSE in a considerable proportion of patients, offering a potential therapeutic option for this critical condition [4].

## Case presentation

An 18-year-old female patient presented with fever, autonomic symptoms, cognitive disorders, and a possible seizure. She was initially managed at another institution with a diagnosis of left ovarian teratoma and subsequently treated under the suspicion of a neuroinfection. After 33 days without clinical improvement and with worsening neurological function and decreased alertness, she was transferred to our hospital. During ambulance transport, she experienced an episode of SE and received immediate management in the emergency department. Her seizure semiology was right-sided head and eye deviation and tonic posture of all four limbs. Following failed treatment for SE, she was administered anesthetizing anti-seizure medications (midazolam (MDZ) 10 mg and brivaracetam (BRV) 400 mg) and was admitted to the ICU. EEG monitoring showed non-convulsive SE.

Due to the age at which our patient presented the ovarian teratoma as well as accompanying symptoms such as autonomic instability, fever, general fatigue, seizures, decreased level of consciousness, speech dysfunction, and rapid onset (<3 months), we suspected anti-NMDA encephalitis based on the diagnostic criteria; therefore, we decided to perform a lumbar puncture to exclude other causes of encephalopathy.

A lumbar puncture was performed, and anti-NMDA antibodies were detected, confirming the diagnosis of anti-NMDA receptor encephalitis (ANRE). MRI showed no abnormalities, and PET/CT with an abnormal brain metabolic pattern suggested autoimmune encephalopathy. She was treated with methylprednisolone, five sessions of plasmapheresis, cyclophosphamide, and seven sessions of electroconvulsive therapy, with no remission of SRSE.

The case was presented at our institution's epilepsy board meeting, and VNS placement was decided. The VNS was implanted, and immediately after closing the skin, under analgesia provided by the neuroanesthesia department (fentanyl), stimulation was turned on with parameters set to normal mode: 1 mA, frequency 30 Hz, duty cycle (DC) 10%, AutoStim 1.25 mA, and magnet mode 1.5 mA. Eleven days post-implantation, the SRSE resolved, and she was discharged from the ICU 75 days later. However, she ultimately died due to her multiple comorbidities (Table 1).

**Table 1:** Patient characteristics N: normal; AS: AutoStim; M: magnet; Freq: frequency

Patient Characteristics	
Sex	Female
Age	18 years old
Duration under anesthetizing anti-seizure medications before VNS implantation	40 days
VNS parameters	N: 1 mA; AS: 1.25 mA; M: 1.5 mA; Freq: 30 Hz; Imp: 500 msec
Neuroanesthesia	Fentanyl
Remission days	11 days
Current status	Died due to her multiple comorbidities

## Discussion

ANRE is a treatable autoimmune disorder characterized by prominent neuropsychiatric symptoms, predominantly affecting children and young adults [5]. The cause of paraneoplastic and autoimmune limbic encephalitis has been recently identified, although its prevalence is likely underestimated. This condition commonly requires admission to the intensive care unit [6].

The treatment of ANRE depends on its etiology. In cases associated with tumors such as ovarian teratomas, prompt tumor removal is crucial. Alongside tumor resection, immunotherapy (intravenous immunoglobulin, PLEX, or corticosteroids) forms the cornerstone of ANRE treatment [3].

The treatment of ANRE-related SE lacks a standardized protocol due to the rarity and complexity of the disease [7]. Second-line immunotherapy options include cyclophosphamide or rituximab. Additionally, electroconvulsive therapy was used in some patients with severe catatonia [8]. Occasionally, ANRE can result in SRSE; however, few cases have been reported in the current scientific literature [6,9,10]. Consequently, other case reports suggest that VNS represents a potential treatment option for seizure control in this pathology, as VNS has altered the natural history of SE [9]. Nevertheless, an optimal treatment is not standardized.

In a non-urgent setting, VNS becomes optimal around the sixth month of treatment, resulting in a 50%-100% reduction in seizure frequency in approximately 45%-65% of the patients (Table 2).

**Table 2:** Differences between activation and therapeutic times according to the type of seizure presentation VNS: vagus nerve stimulation; SRSE: super-refractory status epilepticus

Presentation Type	VNS Activation	Time to Reach Therapeutic Parameters
Elective (drug-resistance epilepsy)	2 weeks postoperative	3 months
Emergencies	24-36 hours postoperative	1 week
SRSE	20 minutes postoperative	20 minutes

However, there is a scarcity of literature regarding the resolution of SRSE using VNS. A systematic review by Furlanis et al. showed that 74% of RSE and SRSE in 38 acute VNS implantations in both adult and pediatric patients were successful in terminating the status [11].

In our case, due to the severity of the patient's condition and before multiple interventions and treatment, with the intention of trying to avoid further neuronal damage, we decided to perform immediate postoperative activation of the VNS based on previously published case reports that demonstrated that it is a safe and effective technique, although there are no standard parameters in the setting of an emergency.

## Conclusions

This case report has shown that VNS titrated to high stimulation parameters immediately postoperatively under specialized neuroanesthesia is safe in treating nonconvulsive SRSE (NCSE) for anti-NMDA receptor limbic encephalitis. We propose that VNS could serve as an effective treatment for NCSE in patients with ANRE who have not responded to other therapies. However, we considered it necessary to investigate the management of ANRE-related NCSE further, extend the existing evidence regarding this approach, and establish it as a viable treatment option in these circumstances, thereby improving the prognosis and reducing the mortality of these patients.
